# Editorial: Nutrients, neurotransmitters and brain energetics, volume II

**DOI:** 10.3389/fnins.2025.1621811

**Published:** 2025-06-10

**Authors:** Rubem Carlos Araújo Guedes, Adriana Ximenes-da-Silva, Daniel C. Anthony

**Affiliations:** ^1^Department of Nutrition, Federal University of Pernambuco, Recife, Brazil; ^2^Department of Physiology, Federal University of Alagoas, Maceió, Brazil; ^3^Department of Pharmacology, University of Oxford, Oxford, United Kingdom

**Keywords:** nutrient, redox imbalance, neurotransmitter receptor, behavior, brain electrophysiology, brain metabolism

## 1 Introduction

The extraordinary energy demands of the brain make it uniquely sensitive to nutritional and metabolic influences. From early development through to late-life neurodegeneration, nutrient availability and metabolic state profoundly shape neuronal excitability, synaptic plasticity, and the integrity of brain networks. It is increasingly evident that these relationships are bidirectional: neurotransmitter systems and cellular energetics are not only regulated by dietary factors but also actively modulate how the brain responds to physiological and environmental challenges. Yet, despite a growing body of evidence linking diet and metabolism to brain function, the underlying mechanisms remain incompletely defined, particularly in relation to neurodegenerative and neuropsychiatric conditions.

This Research Topic brings together a multidisciplinary collection of articles examining the intersections between nutrition, neurotransmission, and brain energetics. The contributions span animal models, human studies, and bioinformatic analyses, and together offer insight into how specific dietary components, neuromodulatory pathways, and metabolic regulators influence brain health. Although diverse in scope, the studies share a central theme: that metabolism is not simply a background process but a dynamic determinant of brain function and dysfunction.

The issue is organized around three interconnected themes. Firstly, studies on nutrition explore the roles of vitamins, fatty acid profiles, caloric restriction, and ketogenic diets in shaping cognitive outcomes, emotional regulation, and neuroprotection. Secondly, the interface between nutrition and neurotransmission is examined through investigations into melatonin signaling, yoga-based neuromodulation, and acupuncture-induced molecular cascades. Thirdly, a set of studies focused on brain energetics highlights how mitochondrial metabolism, redox balance, and network-level connectivity are influenced by dietary and metabolic interventions.

Together, these studies illustrate the breadth of current approaches to understanding how food, metabolism, and signaling converge in the brain. They underscore the need for integrated models that span molecular, cellular, and systems-level domains — and they point toward promising avenues for developing personalized nutritional and metabolic strategies to support cognitive resilience and prevent neurological disease.

## 2 Studies on the subtopic “Nutrition”

A number of studies in this Research Topic underscore the importance of dietary components in shaping cognitive function and vulnerability to neurological disease. The association between vitamin status and brain health is particularly well-illustrated by two large-scale epidemiological studies. Zhang et al. (also see Section 4) report that higher dietary intake of niacin—a precursor of the essential redox coenzyme NAD^+^—was associated with a significantly lower risk of Parkinson's disease in US adults, with each 10 mg increase in niacin intake linked to a 23% risk reduction. Similarly, Pan et al. demonstrate a robust inverse association between serum 25-hydroxyvitamin D levels and cognitive frailty in older adults, with a 12% reduction in frailty risk per unit increase in 25-(OH)D. Together, these findings suggest that even modest differences in micronutrient intake or status may confer meaningful protection against neurodegeneration and age-related cognitive decline.

In contrast to these beneficial effects, Wang et al. highlight a potentially adverse nutritional signal: elevated levels of plasma polyunsaturated fatty acids (PUFAs), and in particular a high arachidonic acid to docosahexaenoic acid (AA/DHA) ratio, were associated with increased depressive symptoms in US adults. These data support the idea that the balance—not just the quantity—of dietary fats may be critical for maintaining mental health and further suggest that dietary lipid profiles could serve as modifiable risk factors in mood disorders.

Experimental studies in rodents provided mechanistic insights into how nutrition influences brain function at both cellular and systems levels. van Rooij et al. (also see Section 4) investigated the effects of caloric restriction (CR) and resveratrol supplementation—a proposed CR mimetic—on resting-state functional connectivity using fMRI in male and female rats. Both interventions altered large-scale brain network activity, with a striking sex-specific effect: reduced functional connectivity between key subcortical and cortical regions was observed predominantly in females. These findings raise important questions about sex differences in metabolic responses and their downstream effects on brain architecture and function.

Finally, the neuroprotective potential of dietary interventions in pathological states was explored by Granados-Rojas et al. (also see Section 4), who examined the impact of a ketogenic diet (KD) in a rodent model of epilepsy. KD feeding preserved expression of the chloride transporter KCC2 in hippocampal subregions, counteracting the reduction induced by seizure activity and shortening after-discharge durations. This suggests that diet-induced shifts in brain energy substrates may not only enhance mitochondrial efficiency but also modulate key ion transport mechanisms critical to neural excitability.

Together, these studies provide compelling evidence that dietary patterns and nutrient composition have profound effects on brain function—from modulating network connectivity in healthy individuals to altering cellular mechanisms in disease. They collectively reinforce the view that targeted nutritional strategies may offer accessible, low-risk interventions to support brain health across the lifespan.

## 3 Studies on the interface “Nutrition and Neurotransmitters”

Cell excitability and transmitter actions were examined through multidisciplinary approaches to evaluate the potential therapeutic effects of melatonin administration, yoga practice and acupuncture stimulation, on behavioral, electrophysiological, and biochemical parameters.

Melatonin is a neurohormone that plays a crucial role in regulating sleep and the circadian rhythm. Additionally, it exhibits a wide range of effects, including blood pressure regulation, antioxidant and anti-inflammatory properties, alongside neuroprotective effects.

Araújo et al. examined in rats the effects of administering two different doses of melatonin on behavioral and electrophysiological parameters of cortical spreading depression (CSD), and redox balance status during brain development.

Animal groups that received a low dose of melatonin (10 mg/kg) exhibited reduced anxiety levels, as measured by the open field and elevated plus maze tests. Both melatonin doses (10 and 40 mg/kg, respectively) influenced brain electrophysiological parameters, with the lower dose significantly decelerating and the higher dose accelerating CSD propagation velocity. Lower malondialdehyde levels and higher superoxide dismutase levels were observed in the cerebral cortex of the group that received the low dose of melatonin. This study highlighted the importance of melatonin's dose-dependent effects on behavior, brain excitability, and redox balance throughout development, and corroborates with the findings from studies with different doses of other antioxidant molecules (Mendes-da-Silva et al., 2014).

Reduced anxiety levels are commonly associated with yoga practice. In this Research Topic, Li et al. investigated in humans the potential effects of yoga practice on reducing anxiety levels through a study assessing the impact of breathing exercises, postures, and mindfulness meditation on brain activity in the prefrontal cortex (PFC).

The study revealed distinct differences in PFC activation between long-term yoga practitioners (>3 times/week for 6.05 years) and short-term practitioners (>3 times/week for 0.91 years). Long-term practitioners exhibited increased oxygenated hemoglobin concentration in the dorsolateral prefrontal cortex, along with enhanced cognitive and emotional regulation. These findings highlight the potential benefits of long-term yoga practice in promoting cognitive improvement and reducing anxiety.

In recent years, several studies have focused on elucidating the cellular mechanisms underlying the physiological effects induced by acupuncture stimulation. Acupoint catgut embedding (ACE) therapy is based on traditional acupuncture techniques and involves the application of absorbable catgut at acupoints. This technique provides prolonged stimulation of acupoints compared to traditional methods, which can be especially beneficial in the treatment of chronic conditions.

Hou et al. investigated in rats the role of mechanically sensitive transient receptor potential vanilloid (TRPV) channels, including TRPV2 and TRPV4, in the regulatory pathways of ACE therapy. Their findings revealed stimulation effects resulting in a physico-chemical-immune response mediated by TRPV channels, calcium influx, and the activation of macrophage CD68 and mast cell tryptase, providing valuable insights into the cellular mechanisms underlying ACE therapy.

## 4 Studies on the interface “Nutrition and Brain Energetics”

Energy metabolism is fundamental to brain function, underpinning processes from ion homeostasis and neurotransmission to large-scale network activity. While not always explicitly framed in terms of energetics, several of the contributions to this Research Topic shed important light on how metabolic interventions and nutritional factors shape neural activity and resilience. A particularly clear mechanistic insight came from the work of Granados-Rojas et al., who examined the effects of a ketogenic diet (KD) in a well-established rodent model of epilepsy. Beyond its recognized role in reducing seizure frequency, KD selectively preserved expression of the chloride transporter KCC2 and shortened after-discharges in hippocampal regions, counteracting the downregulation induced by amygdala kindling. Higher KCC2 levels are linked to shorter generalized seizures, explaining the KD's beneficial effect on epilepsy. Since KCC2 is essential for maintaining inhibitory synaptic transmission, its preservation likely reflects improved cellular energetics and ionic homeostasis under ketotic conditions. Notably, KCC2 levels correlated inversely with after-discharge duration, suggesting that diet-driven shifts in brain metabolism can have direct functional consequences for excitability.

At the level of brain networks, van Rooij et al. demonstrated that both caloric restriction (CR) and the CR-mimetic resveratrol modulated resting-state functional connectivity (FC) in rats, with pronounced and sex-specific effects. In females, both interventions reduced connectivity between key subcortical and cortical regions, including the hippocampus. These findings raise intriguing questions about the relationship between systemic metabolic state, neurovascular coupling, and network dynamics. The authors discuss the possibility of the vascular contribution to the BOLD signal in the context of their interventions, which seemed very interesting to us. This study's insight could be considered a functional connectivity reference for further investigation. Given the energy demands of maintaining synchronized neural activity, interventions that reshape connectivity may ultimately act, at least in part, via modulation of brain energetics.

Perhaps unsurprisingly, mitochondrial metabolism emerged as a recurring theme. Qin et al., using a bioinformatics approach, identified key genes involved in acetyl-CoA synthesis, mitochondrial respiration and pyruvate metabolism that may serve as biomarkers of cuproptosis in cerebral ischemia. These processes are central to cellular energy metabolism, linking nutrient oxidation to ATP generation. Their findings further underscore the critical role of mitochondrial integrity in brain injury and repair.

Finally, dietary micronutrients with recognized roles in redox and energy metabolism were linked to neurodegenerative disease risk. Zhang et al. found that higher dietary niacin intake—a precursor of NAD+, a vital coenzyme in mitochondrial metabolism—was inversely associated with Parkinson's disease prevalence in a large US cohort, with a 23% reduction in risk for each 10 mg increase in niacin intake This raises the possibility that even subtle nutritional deficits may impair brain energetics over the lifespan, contributing to neurodegeneration.

These findings align with our own previous observations that systemic metabolic state and peripheral inflammation can influence brain metabolism, including in the context of neurodegenerative and neuropsychiatric conditions (Dunstan et al., 2024; Aziz et al., 2025). In particular, our work has highlighted the interaction between metabolic substrates, inflammatory mediators, and astrocyte-neuron coupling (Radford-Smith et al., 2024) as a critical determinant of brain energy homeostasis—a theme echoed in several of the contributions to this Research Topic.

Together, the studies reviewed here highlight the rich interplay between diet, metabolism, and brain energetics. While mechanisms range from the cellular to the network level, all emphasize the potential of nutritional and metabolic interventions to modulate brain function and potentially ameliorate disease processes.

## 5 Concluding remarks

In conclusion, the studies published in this Research Topic represent an important contribution to our evolving understanding of how nutrition, neurotransmitters and brain energetics interact to shape brain function across the lifespan. Although diverse in approach and focus, together they highlight the intricate ways in which metabolic and nutritional factors can influence neuronal excitability, network connectivity, and ultimately, behavior and cognition.

In the nutrition theme, several of the studies underscored the protective or deleterious roles of dietary components on brain health. While higher intake of vitamins such as niacin and vitamin D was associated with reduced risk of Parkinson's disease and cognitive frailty, respectively, other findings highlighted potential risks of imbalanced dietary fat composition. The study of polyunsaturated fatty acids revealed that an elevated AA/DHA ratio may increase susceptibility to depression, illustrating how nutritional imbalances may perturb brain function and mental health.

In the neurotransmitters and neuromodulation section, multidisciplinary approaches revealed that targeted interventions such as melatonin supplementation, yoga, and acupuncture can modulate anxiety, excitability and redox status. These findings point to a key role for neuromodulatory systems in mediating the effects of lifestyle and therapeutic interventions on brain activity. Of particular interest is the evidence that both low-dose melatonin and long-term yoga practice exert anxiolytic and cognitive benefits, mediated through alterations in cortical excitability and prefrontal cortex function.

Under the Brain Energetics theme of volume II, while not all contributions addressed this subtopic explicitly, many provided critical insights. Studies demonstrated that metabolic interventions—from ketogenic diets and caloric restriction to micronutrient supplementation—can reshape brain function through mechanisms ranging from mitochondrial modulation and cuproptosis-related pathways to altered functional connectivity. Dietary modulation of substrates and redox cofactors, as seen with niacin intake and KCC2 regulation, further supports the view that brain energetics is a crucial integrative axis linking nutrition and neuronal physiology. These observations are in keeping with emerging work suggesting that systemic metabolic state, peripheral inflammation and astrocyte-neuron metabolic coupling together determine energetic resilience in the brain.

Taken together, the contributions in this Research Topic reflect a growing recognition of the importance of integrating molecular, cellular, and systems-level perspectives in the study of brain energetics and its nutritional and neurochemical determinants. The multidisciplinary approaches presented here—ranging from human cohort studies to animal models and bioinformatics—provide valuable platforms for future research aimed at developing novel metabolic and nutritional strategies to promote brain health and prevent or mitigate neurodegenerative and neuropsychiatric disorders.
